# CYR61 Acts as an Intracellular Microtubule-Associated Protein and Coordinates Mitotic Progression via PLK1-FBW7 Pathway

**DOI:** 10.7150/ijbs.93335

**Published:** 2024-06-03

**Authors:** Kaishun Hu, Limin Xie, Wenjing Wu, Jingyuan Zhang, Yu Li, Jiehua He, Yin Zhang, Daning Lu, H. Phillip Koeffler, Lehang Lin, Dong Yin

**Affiliations:** 1 Guangdong Provincial Key Laboratory of Malignant Tumor Epigenetics and Gene Regulation, Guangdong-Hong Kong Joint Laboratory for RNA Medicine, Medical Research Center, Sun Yat-Sen Memorial Hospital, Sun Yat-Sen University, Guangzhou 510120, P.R. China.; 2 Department of Breast Oncology, Sun Yat-Sen Memorial Hospital, Sun Yat-Sen University, Guangzhou 510120, P.R. China.; 3 Department of Laboratory Medicine, Peking University Shenzhen Hospital, Shenzhen 518000, P.R. China.; 4 Cancer Science Institute of Singapore, National University of Singapore, 117599, Singapore.; 5 Department of Medicine, Cedars-Sinai Medical Center, Los Angeles, CA 90048, USA.

**Keywords:** CYR61, PLK1, mitosis, microtubules, FBW7

## Abstract

Cysteine-rich angiogenic inducer 61 (CYR61), also called CCN1, has long been characterized as a secretory protein. Nevertheless, the intracellular function of CYR61 remains unclear. Here, we found that CYR61 is important for proper cell cycle progression. Specifically, CYR61 interacts with microtubules and promotes microtubule polymerization to ensure mitotic entry. Moreover, CYR61 interacts with PLK1 and accumulates during the mitotic process, followed by degradation as mitosis concludes. The proteolysis of CYR61 requires the PLK1 kinase activity, which directly phosphorylates two conserved motifs on CYR61, enhancing its interaction with the SCF E3 complex subunit FBW7 and mediating its degradation by the proteasome. Mutations of phosphorylation sites of Ser167 and Ser188 greatly increase CYR61's stability, while deletion of CYR61 extends prophase and metaphase and delays anaphase onset. In summary, our findings highlight the precise control of the intracellular CYR61 by the PLK1-FBW7 pathway, accentuating its significance as a microtubule-associated protein during mitotic progression.

## Introduction

Cysteine-rich angiogenic inducer 61 (CYR61), also named CCN1, is one of six members of the CCN family which is characterized by a high degree of amino acid sequence homology ranging from 50~90% 1, 2. Recently, studies have shown that CYR61 is essential for multiple physiological processes, including cardiovascular development, tissue repair, and inflammation regulation 3-5. Meanwhile, abnormal CYR61 expression is associated with pathological abnormalities including cancers 6-12. Acting as a ligand, the various functions of secreted CYR61 depend on its binding with distinct integrins. For example, CYR61 can either promote cell proliferation, migration, and survival by binding to αvβ3 integrin, or induce apoptosis and senescence through integrin α6β1 and heparin sulfate proteoglycans 13. Although being widely acknowledged as a secretory and matricellular protein, CYR61 is also intracellularly localized and has been detected in the cell nucleus 14, 15. However, the intracellular roles of CYR61 are not well understood.

Cell cycle progression, which typically comprises four phases, G1, S, G2, and M, is a complex sequence of events that drives a cell to divide and produce two new daughter cells 16. Mitosis (M phase) is an important event of the cell cycle because it is the most dynamic and fragile period. As such, mitosis becomes a frequent target for cancer therapy, with a majority of cancer medications being designed to specifically address mitotic cells or mitotic regulators such as cyclin-dependent kinases (CDKs), Aurora kinases, and polo‑like kinases (PLKs) 17. Therefore, maintaining the stability of mitosis is of great significance to lives and organisms.

In order to maintain the consecutive progression of the cell mitosis, two essential E3 ubiquitin ligase complexes play crucial roles: the anaphase-promoting complex/cyclosome (APC/C) and the S-phase kinase-associated protein 1 (SKP1)-cullin 1 (CUL1)-F-box protein complex (SCF). These ubiquitin-protein ligases target specific cell cycle regulatory proteins for degradation via the proteosome pathway 18, 19. For example, the APC/C is a large multi-subunit cullin RING E3 ubiquitin ligase that facilitates the ubiquitination of securin, an inhibitor of anaphase, thus promoting the transition from metaphase to anaphase. Meanwhile, APC/C also controls the exit from mitosis and maintenance of G1 by ubiquitinating mitotic cyclins 20, 21. On the other hand, the SCF complex consists of four components: the linker protein SKP1, the RING finger protein RBX1, the scaffold protein CUL1, and a variable subunit known as the F-box protein. In the human genome, there are 69 F-box proteins identified, which serve as the recognition subunit of the SCF complex and determine the specificity for SCF substrates 22. Recent study has proved that the SCF-FBW7 E3 ubiquitin ligase prevents mitotic slippage through triggering the degradation of WDR5, a component of the histone 3 lysine 4 methyltransferase complex 23.

In this study, we mainly explored the biological functions and regulatory mechanisms of CYR61 inside cells. We proved that both the timely accumulation and exquisite degradation of CYR61 are crucial for cell mitotic progression. Mechanistically, during the early mitosis, CYR61 regulates microtubule polymerization to ensure the proper onset of mitotic entry. Furthermore, CYR61 interacts with PLK1 and accumulates in the mitotic phase, followed by its own degradation in both PLK1‑ and FBW7-dependent manners. Notably, retarded protein degradation of CYR61 prolongs prophase and metaphase and delays anaphase onset, resulting in a state of mitotic arrest. Taken together, our findings lead to the conclusion that CYR61 acts a novel microtubule-associated protein, which regulates mitosis and serves as a potential target for cancer treatment.

## Results

### CYR61 interacts with and stabilizes microtubules

**Figure 1:**
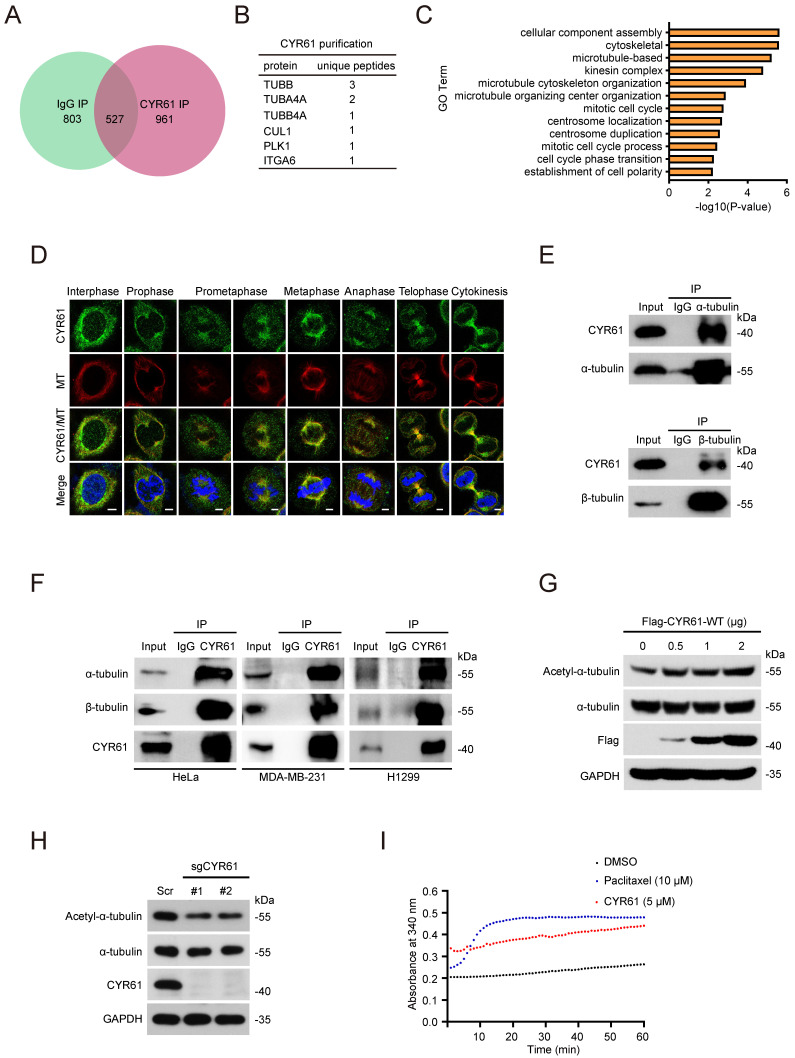
CYR61 interacts with tubulins and promotes microtubule assembly and stability. **(A and B)** Venn diagram (A) and chart (B) illustrating CYR61-associated proteins identified by mass spectrometry. **(C)** Gene Ontology (GO) enrichment analysis of CYR61-associated proteins identified by mass spectrometry. **(D)** Co-localization of endogenous CYR61 and microtubule (MT) during the mitotic process. HeLa cells were co-stained with anti-CYR61 (green) and anti-β-tubulin (red) antibodies. DAPI (blue) was used to stain the compact chromosomes. Scale bars represent 2 μm. **(E)** Cell lysates from HeLa cells were immunoprecipitated with either anti-α/β-tubulin antibodies or IgG and were subsequently immunoblotted using indicated antibodies. Biological triplicates were performed and the representative results are presented. **(F)** Cell lysates from HeLa, MDA-MB-231, and H1299 cells were immunoprecipitated with either anti-CYR61 antibody or IgG and were subsequently immunoblotted using indicated antibodies. Biological triplicates were performed and the representative results are presented. **(G)** HeLa cells transfected with the indicated amounts of plasmids were lysed with RIPA buffer and analyzed by western blotting using indicated antibodies. Biological triplicates were performed and the representative results are presented. **(H)** HeLa cells transfected with either Scramble (Scr) or *CYR61* sgRNAs (#1 and #2) were lysed with RIPA buffer and analyzed by western blotting using indicated antibodies. Biological triplicates were performed and the representative results are presented. **(I)** Microtubule assembly was documented at 37 ℃ in the presence of DMSO, paclitaxel (10 μM), or *in vitro* translated CYR61 (5 μM).

Our previous studies have proved the crucial roles of CYR61 in cancer proliferation and migration 2, 24, 25. In order to gain deeper insights into the involvement of CYR61 in intracellular regulation, we performed tandem affinity purification coupled with mass spectrometry (MS) analysis to identify proteins that potentially interact with CYR61 (Figure 1A, B). Employing Gene Ontology (GO) analysis through the CPDB database (http://cpdb.molgen.mpg.de/), we revealed that proteins interacting with CYR61 are significantly enriched in biological processes related to the mitotic cell cycle, including cellular component assembly, microtubule-based, microtubule cytoskeleton organization, centrosome localization and duplication, and cell cycle phase transition, etc. (Figure 1C). Importantly, we also made a novel discovery of tubulin as a binding partner for CYR61 (Figure 1B and Supplementary Table 1). These findings suggest the potential engagement of CYR61 in the process of cell mitosis.

To confirm the interaction between tubulin and CYR61, we conducted immunofluorescence assays, which demonstrated the co-localization of endogenous CYR61 with microtubules throughout the entire mitotic process (Figure 1D). Meanwhile, through immunoprecipitation (IP) assays, we clearly detected CYR61, α-tubulin, and β-tubulin in the immunoprecipitated complex from various cancer cell lines (Figure 1E, F). IP assays also indicated the interactions between CYR61 and other tubulin-associated proteins, such as DVL-1, DVL-2, DVL-3, NUMA, and DIC1/2 (Figure S1).

As is widely understood, the polymerization of α- and β-tubulin dimers forms microtubules, which are main components of the mitotic spindle and crucial for cell division 26. Hence, we also determined the role of CYR61 on tubulin polymerization. As shown in Figure 1G, the overexpression of Flag-tagged CYR61 in HeLa cells led to a dose-dependent increase in acetylated α-tubulin, a marker of microtubule stability 27. Conversely, depletion of CYR61 remarkably decreased the level of acetylated α-tubulin (Figure 1H), indicating that CYR61 promotes microtubule assembly. We then assessed the process of the assembly of tubulin subunits into microtubules in an *in vitro* setting. Through monitoring dynamic changes of tubulin turbidity at 37 °C, we demonstrated that CYR61 was able to enhance the tubulin polymerization, albeit its effect was not as pronounced as that of paclitaxel (Figure 1I). These results suggest that CYR61 has the capability to facilitate tubulin polymerization into microtubules.

### CYR61 is important for the mitotic process

**Figure 2:**
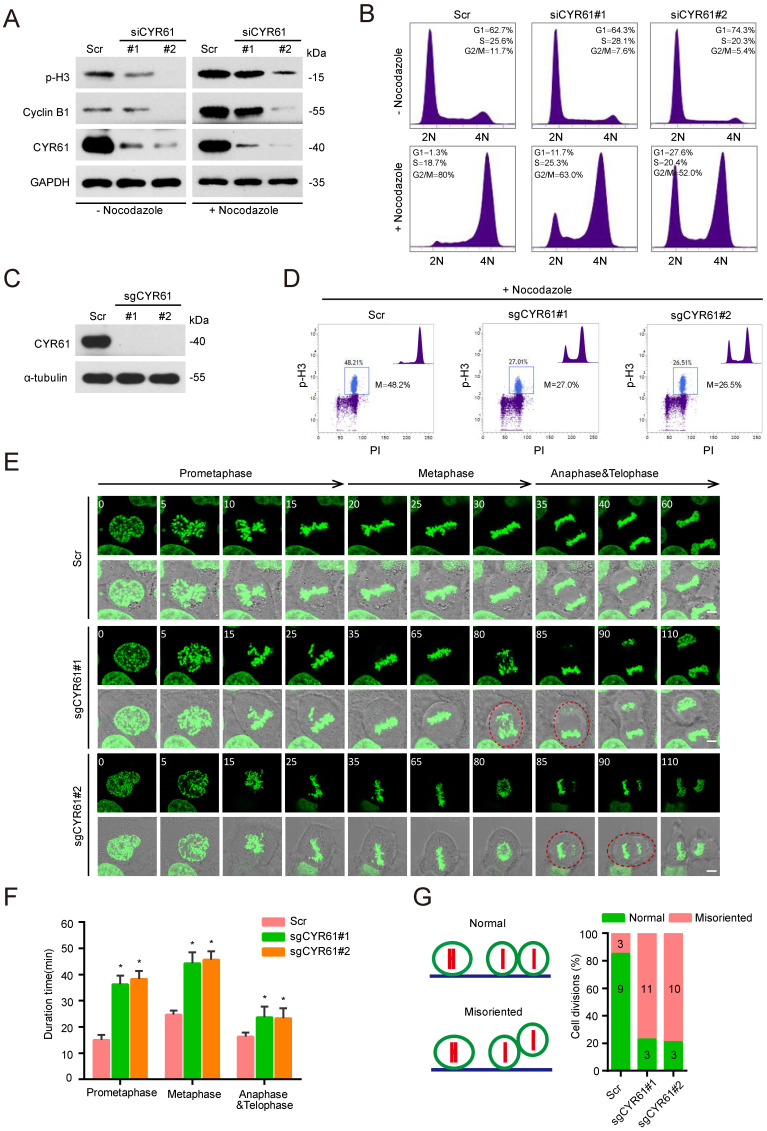
CYR61 is important for the mitotic process. **(A)** HeLa cells transfected with either Scramble (Scr) or *CYR61* siRNAs (#1 and #2) were incubated in the presence or absence of nocodazole (100 ng/ml, 12 h). Subsequently, cells were lysed with RIPA buffer and analyzed by western blotting using indicated antibodies. Biological triplicates were performed and the representative results are presented. **(B)** HeLa cells transfected with either Scramble (Scr) or *CYR61* siRNAs (#1 and #2) were incubated in the presence or absence of nocodazole (100 ng/ml, 12 h) and analyzed by flow cytometry. Biological duplicates were performed and the representative results are presented. **(C)** HeLa cells transfected with either Scramble (Scr) or *CYR61* sgRNAs (#1 and #2) were lysed with RIPA buffer and analyzed by western blotting using indicated antibodies. Biological triplicates were performed and the representative results are presented. **(D)** HeLa cells transfected with either Scramble (Scr) or *CYR61* sgRNAs (#1 and #2) were incubated in the presence of nocodazole (100 ng/ml, 12 h) and analyzed by flow cytometry. The percentage of mitotic cells, as indicated by the staining for phospho-histone H3 (p-H3), is reported. Biological duplicates were performed and the representative results are presented. **(E)** The kinetics of mitotic progression in HeLa/GFP-H2B cells were analyzed through time-lapse microscopy, which showed that the depletion of CYR61 prolongs both prometaphase and metaphase progression. Scr represents scramble; Scale bars represent 2 μm; The time unit is minutes. **(F and G)** The quantification of mitotic progression durations (F) and normal or misoriented cell divisions (G) for each cell model in panel (E). Fifteen cell images were captured from each cell model; Scr represents scramble; Error bars indicate SEM; *, *P* < 0.05; *P* values were calculated using the Student's t-test.

Next, we performed a cell synchronization assay using nocodazole treatment to block HeLa cells in the early mitosis and evaluated the potential impact of CYR61 on cell cycle progression. Of note, we observed that either in the presence or absence of nocodazole treatment, knockdown of endogenous CYR61 was accompanied by the decreased protein levels of Cyclin B1 and phospho-histone H3 (p-H3), two classic mitotic markers (Figure 2A). Flow cytometry assays also reflected that CYR61 depletion significantly reduced the percentage of cells in the M phase (Figure 2B and Figure S2A). These findings suggest that CYR61 is crucial for the mitotic entry. Additionally, this deficiency in mitotic entry was recapitulated using single guide RNAs (sgRNAs) targeting *CYR61* (Figure 2C, D and Figure S2B) and could be successfully reversed in cells reintroduced with wild-type (WT) CYR61 (Figure S2C, D), ruling out the possibility that our observations were due to small interfering RNA (siRNA) off-target effects. Furthermore, the progression of mitotic exit was profoundly delayed in cells after depletion of CYR61 (Figure S2E). Collectively, these data indicate that CYR61 is essential for both mitotic entry and progression.

For further visualizing the specific function of CYR61 during the mitotic process, we established a stable HeLa cell line that constitutively expresses a fusion protein combining histone 2B and green fluorescent protein (H2B-GFP). This system allows the high-resolution imaging of chromosome dynamics in living cells 28. Time-lapse observation revealed that various stages of the mitotic process, including the prometaphase, metaphase, anaphase, and telophase, were all significantly prolonged in cells lacking* CYR61* (Figure 2E, F). Moreover, a large proportion of CYR61-depleted cells displayed irregular and uneven timing in terms of daughter cell adhesion to the substratum, suggesting a potential role of CYR61 in ensuring the accurate orientation and positioning of the mitotic spindle (Figure 2E, G).

### CYR61 interacts with PLK1 during mitosis

**Figure 3:**
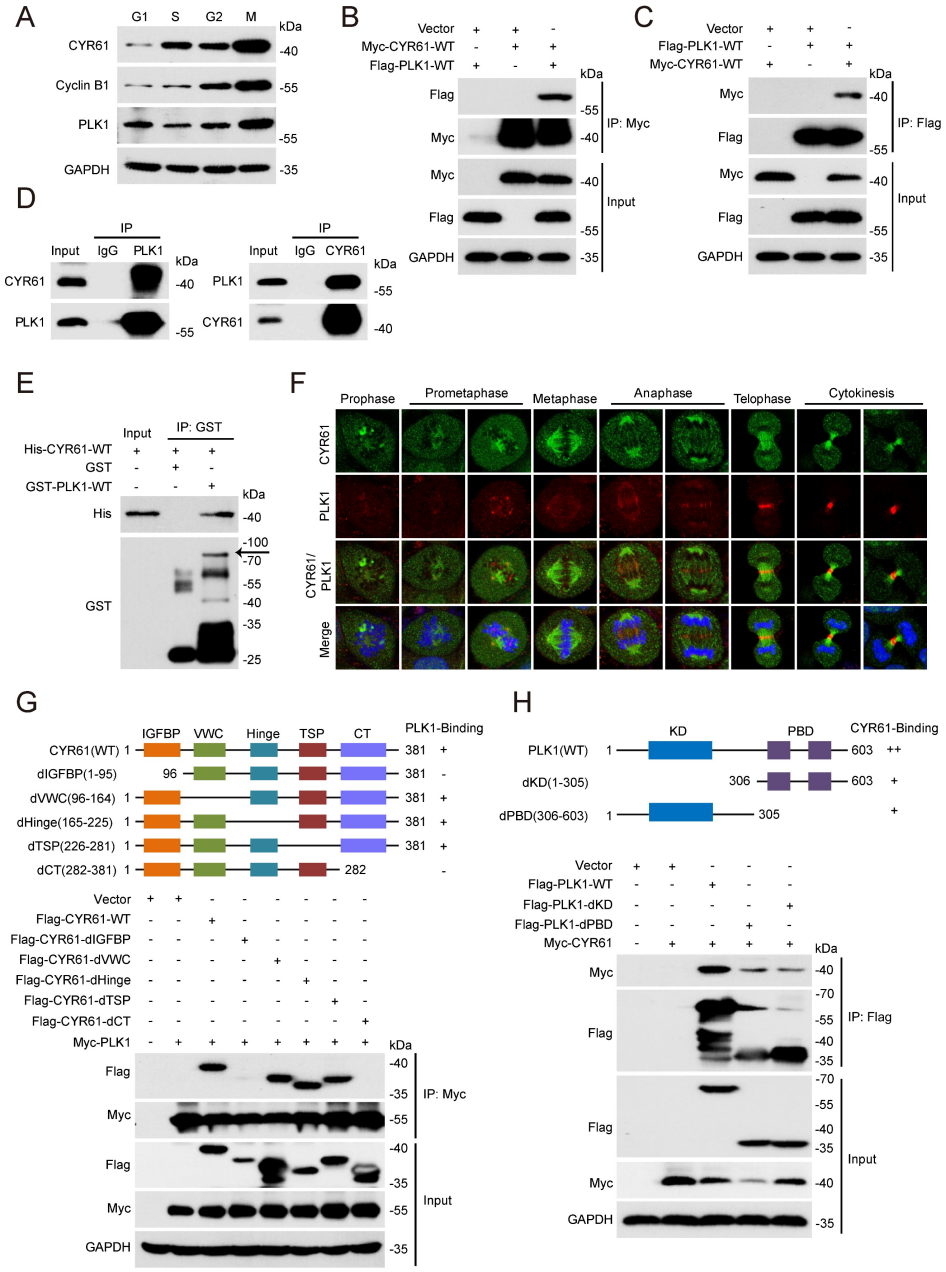
CYR61 interacts with PLK1. **(A)** HeLa cells synchronized using a double thymidine block-and-release method were collected at various phases of the cell cycle, including G1, S, G2, and M, and then lysed with RIPA buffer. Subsequently, cell lysates were subjected to western blotting using indicated antibodies. Biological triplicates were performed and the representative results are presented. **(B and C)** HEK293T cells overexpressing indicated plasmids were subjected to immunoprecipitation using anti-Myc (B) or anti-Flag (C) beads. Subsequently, cell lysates were analyzed by western blotting using indicated antibodies. Biological triplicates were performed and the representative results are presented. **(D)** Cell lysates from HeLa cells were immunoprecipitated with anti-PLK1, anti-CYR61, or IgG, and were subsequently immunoblotted using indicated antibodies. Biological triplicates were performed and the representative results are presented. **(E)** The fusion protein His-CYR61-WT was incubated with either GST or GST-PLK1-WT at 4℃ overnight. Subsequently, anti-GST beads were added into the system, followed by separation and western blotting using indicated antibodies. The arrow denotes the position of GST-PLK1-WT. Biological triplicates were performed and the representative results are presented. **(F)** Co-localization of endogenous CYR61 and PLK1 during the mitotic process. HeLa cells were co-stained with anti-CYR61 (green) and anti-PLK1 (red) antibodies. DAPI (blue) was used to stain the compact chromosomes. Scale bars represent 2 μm. **(G and H)** HeLa cells were transfected with indicated plasmids and then immunoprecipitated using either anti-Myc (G) or anti-Flag (H) beads. Cells lysates were subsequently analyzed by western blotting using indicated antibodies. Biological triplicates were performed and the representative results are presented.

We also measured the expression variation of CYR61 across different cell cycle phases using HeLa cell line model. Notably, we observed that the CYR61 level was remarkably low at the G1 phase, followed by a gradual rise and peaked at the M phase (Figure 3A). This intriguingly mirrored the expression pattern of PLK1, a serine/threonine protein kinase primarily active during mitosis 29 and was also identified in the CYR61 MS results (Figure 1B).

We then employed transient transfection and co-IP experiments to investigate the potential connection between CYR61 and PLK1 *in vitro*. We clearly detected a complex containing CYR61 and PLK1 using either anti-Myc or anti-Flag beads in human embryonic kidney cells (HEK293T) co-expressing Myc-CYR61 and Flag-PLK1 (Figure 3B, C). Secondly, we harvested HeLa whole cell extracts and observed the endogenous interaction between CYR61 and PLK1 (Figure 3D). Importantly, the interaction between CYR61 and PLK1 appeared to be direct, as recombinant His-CYR61 successfully bound with recombinant GST-PLK1 *in vitro* (Figure 3E). We also investigated the spatiotemporal dynamic expression of CYR61 in relative to PLK1 during mitotic progression using immunofluorescence assays. Evidently, the localization of CYR61 shifted successively throughout the mitotic process: from centrosomes to kinetochores, then to microtubules, the midzone, and finally to the midbody. In contrast, PLK1 was localized at centrosomes and kinetochores during prophase to metaphase, and at the midzone and the midbody from anaphase to telophase. These results indicate that CYR61 and PLK1 co-localize at centrosomes, kinetochores, the midzone, and the midbody, respectively, during different stages of mitosis (Figure 3F).

The molecular architecture of CYR61 includes five domains: an insulin-like growth factor binding protein-like module (IGFBP), a von Willebrand factor type C repeat module (VWC), a Hinge module, a thrombospondin type-1 repeat module (TSP-1), and a cysteine-knot-containing module (CT) 30. While PLK1 comprises only two distinguishable domains: the N-terminal kinase domain (KD) for ATP-mediated catalysis and the C-terminal polo-box domain (PBD) for recognizing downstream binding targets or substrates. We further generated a series of CYR61 and PLK1 mutants and mapped the specific co-binding region(s) between them. IP assays using deletion of individual module of CYR61 demonstrated that none of the VWC, the Hinge, and the TSP-1 domains was required for CYR61‑PLK1 interaction, whereas the N-terminal IGFBP and the C-terminal CT domains of CYR61 were both indispensable (Figure 3G). On the other hand, either the KD or the PBD of PLK1 alone was capable of binding with CYR61, albeit at a significantly reduced level compared to WT PLK1 (Figure 3H). These results support that CYR61 physically and specifically interacts with PLK1.

### PLK1 post-translationally regulates CYR61 expression

Normally, PLK1 undergoes phosphorylation at Thr210 by Aurora A and Bora to achieve full activation prior to entering mitosis 31-33. Subsequently, PLK1 directly phosphorylates and facilitates the degradation of various substrates in a cell cycle-dependent manner, thereby influencing mitotic transition events, including sister chromatid separation and cytokinesis 34, 35.

**Figure 4:**
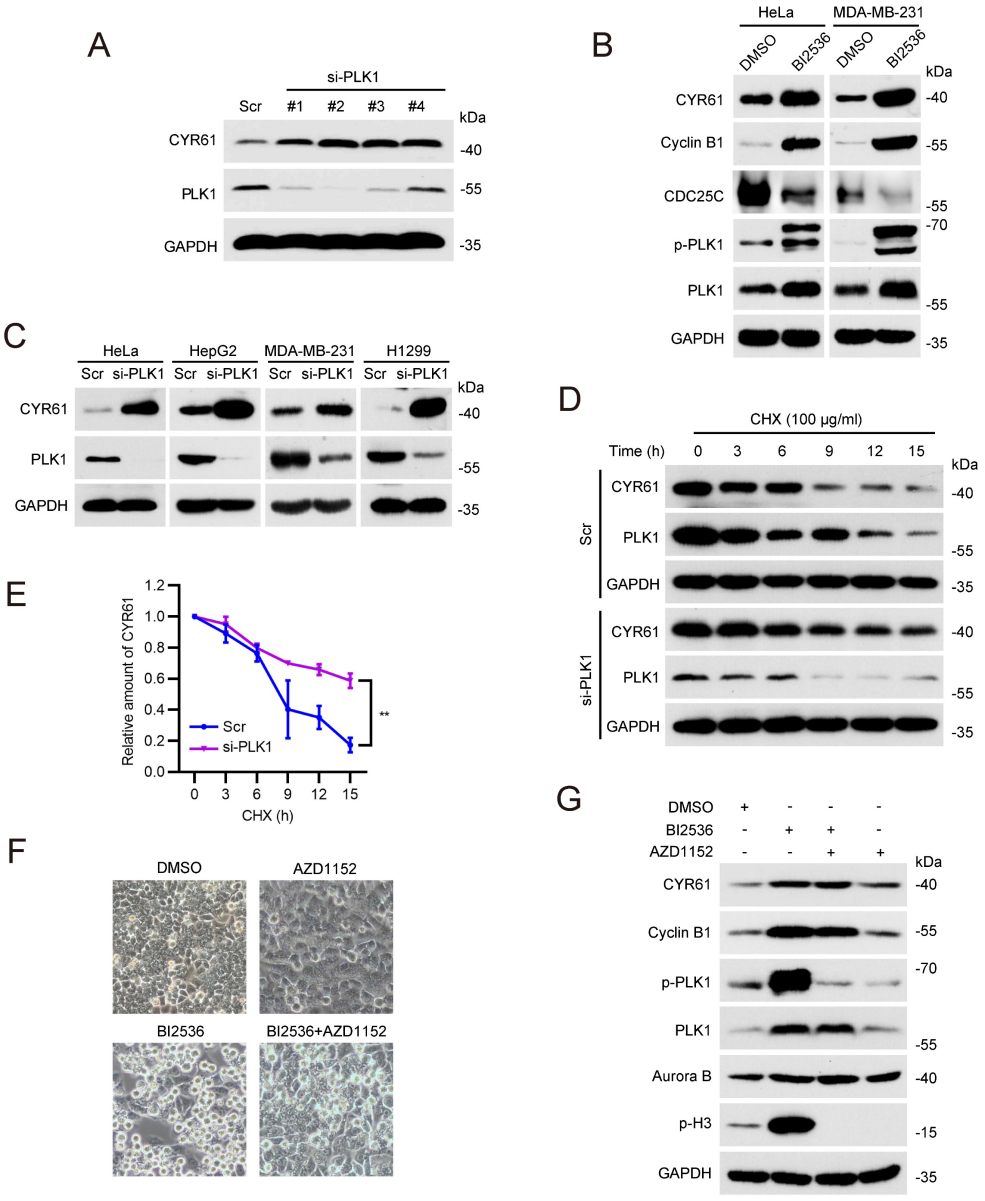
PLK1 promotes the degradation of CYR61. **(A)** HeLa cells transfected with either Scramble (Scr) or *PLK1* siRNAs (#1, #2, #3, and #4) were lysed with RIPA buffer and analyzed by western blotting using indicated antibodies. Biological triplicates were performed and the representative results are presented. **(B)** HeLa and MDA-MB-231 cells treated with either DMSO or BI2536 (10 μM) were lysed with RIPA buffer and analyzed by western blotting using indicated antibodies. Biological triplicates were performed and the representative results are presented. **(C)** HeLa, HepG2, MDA-MB-231, and H1299 cells transfected with either Scramble (Scr) or *PLK1* siRNA were lysed with RIPA buffer and analyzed by western blotting using indicated antibodies. Biological triplicates were performed and the representative results are presented. **(D)** HeLa cells transfected with either Scramble (Scr) or *PLK1* siRNAs were treated with CHX (100 μg/ml) for the indicated periods of time. Subsequently, cells were lysed with RIPA buffer and cell lysates were analyzed by western blotting using indicated antibodies. Biological triplicates were performed and the representative results are presented. **(E)** The quantification of CYR61 protein levels from panel (D). Error bars indicate SEM; **, *P* < 0.01; The *P* value was calculated using the Student's t-test. **(F)** Representative HeLa cell images are shown after indicated treatments. **(G)** HeLa cells undergoing indicated treatments were lysed with RIPA buffer and analyzed by western blotting using indicated antibodies. Biological triplicates were performed and the representative results are presented.

In light of this, we tested whether PLK1 affects the stability of CYR61 during mitosis. We observed that depletion of PLK1 in HeLa cells led to a significant increase in the CYR61 protein level (Figure 4A). Treatment with a specific PLK1 inhibitor, BI2536, also resulted in elevated CYR61 protein expression, while simultaneously increasing Cyclin B1 and decreasing CDC25C, both of which are indicative markers of PLK1 activity inhibition (Figure 4B) 36-38. This negative correlation between CYR61 and PLK1 was further confirmed in multiple cancer cell lines, including HepG2, MDA-MB-231, and H1299 (Figure 4C), and did not occur at the transcriptional level (Figure S3). Importantly, the half-life of endogenous CYR61 significantly increased in PLK1-depleted cells in the presence of cycloheximide (CHX), an inhibitor of protein synthesis (Figure 4D, E). These results imply that PLK1 specifically regulates CYR61 expression at the post-transcriptional level.

It has been documented that either the inhibition or depletion of PLK1 can activate the spindle assembly checkpoint (SAC) and result in profound mitotic arrest 39. We therefore doubted whether the CYR61 accumulation in response to PLK1 inhibition is primarily a regulatory effect of PLK1 or simply a direct outcome of the mitotic arrest. As seen in Figures 4F and 4G, HeLa cells treated with BI2536 alone were arrested in mitosis, as evidenced by increased amounts of spherical cells and elevated levels of Cyclin B1 and p-H3. However, with the addition of the Aurora B inhibitor, AZD1152, the SAC was deactivated, forcing HeLa cells to exit mitosis, which was supported by restored cell shape and the absence of p-H3^40^. Notably, cells treated with both BI2536 and AZD1152 still displayed higher level of CYR61 compared to those treated with AZD1152 alone (Figure 4G). These observations prove that the accumulated CYR61 in response to PLK1 inhibition is indeed a direct consequence of the regulatory role of PLK1.

### PLK1 phosphorylates CYR61 at both Ser167 and Ser188 sites and modulates its degradation

**Figure 5:**
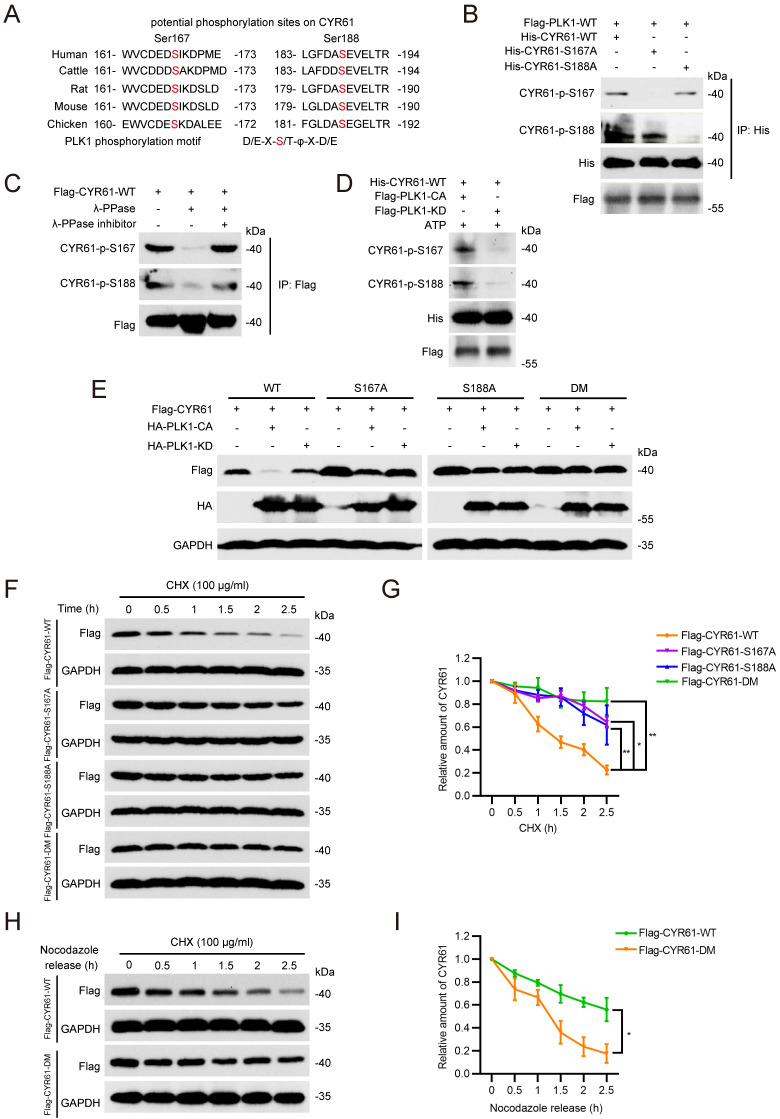
PLK1 phosphorylates CYR61 at the Ser167 and Ser188 sites. **(A)** Potential phosphorylation sites on CYR61 across various species. Sites of Ser167 and Ser188 are highlighted in red. **(B)** HeLa cells transfected with indicated plasmids were immunoprecipitated with anti-His beads. Subsequently, cell lysates were analyzed by western blotting using indicated antibodies. Biological triplicates were performed and the representative results are presented. **(C)** HeLa cells transfected with Flag-CYR61-WT were treated in the presence or absence of lambda phosphatase (λ-PPase) and a λ-PPase inhibitor followed by immunoprecipitated with anti-Flag beads. Cells were lysed with RIPA buffer and analyzed by western blotting using indicated antibodies. Biological triplicates were performed and the representative results are presented. **(D)** His-tagged CYR61-WT protein translated *in vitro* was incubated with either Flag-PLK1-CA or Flag-PLK1-KD. Subsequently, samples were analyzed by western blotting using indicated antibodies. Biological triplicates were performed and the representative results are presented. **(E)** HeLa cells transfected with indicated plasmids were lysed with RIPA buffer and analyzed by western blotting using indicated antibodies. Biological triplicates were performed and the representative results are presented. **(F)** HeLa cells were transfected with indicated plasmids and treated with CHX (100 μg/ml) for the indicated periods of time. Subsequently, cells were lysed with RIPA buffer and analyzed by western blotting using indicated antibodies. Biological triplicates were performed and the representative results are presented. **(G)** The quantification of CYR61 protein levels from panel (F). Error bars indicate SEM; *, *P* < 0.05; **, *P* < 0.01; *P* values were calculated using the Student's t-test. **(H)** HeLa cells transfected with indicated plasmids were treated with nocodazole (100 μg/ml, 12h) and CHX (100 μg/ml) and released for the indicated periods of time. Subsequently, cells were lysed with RIPA buffer and analyzed by western blotting using indicated antibodies. Biological triplicates were performed and the representative results are presented. **(I)** The quantification of CYR61 protein levels from panel (H). Error bars indicate SEM; *, *P* < 0.05; The *P* value was calculated using the Student's t-test.

Next, we sought to determine whether CYR61 functions as a substrate of PLK1. We generated two polyclonal phospho-specific antibodies, referred to as anti-CYR61-p-S167 and anti-CYR61-p-S188, based on two evolutionarily conserved phosphorylation sites of PLK1 on CYR61, Ser167 (S167) and Ser188 (S188) (Figure 5A) 41. Indeed, both CYR61-p-S167 and CYR61-p-S188 were clearly detected in HeLa cells ectopically transfected with WT CYR61 (Figure 5B). Nonetheless, when cells were transfected with the S167A or S188A mutant of CYR61, where the serine residue at position 167 or 188 was replaced with alanine, we failed to detect CYR61-p-S167 and CYR61-p-S188, respectively (Figure 5B). Moreover, the signals of CYR61-p-S167 and CYR61-p-S188 were both susceptible to phosphatase treatment, as they substantially diminished when cell lysates were pre-treated with lambda phosphatase (λ-PPase) (Figure 5C). These results conclusively demonstrate that PLK1 effectively phosphorylates CYR61 at both the S167 and S188 sites. Additionally, the anti-CYR61-p-S167 and anti-CYR61-p-S188 antibodies are proved to be specific in detecting phosphorylation at S167 and S188 in CYR61, respectively.

Using an *in vitro* kinase assay, we also demonstrated that the phosphorylation at both S167 and S188 of CYR61 was dependent on the kinase activity of PLK1, since unlike the catalytically active (CA) PLK1, the PLK1 kinase-dead (KD) mutant lacked the ability to phosphorylate WT CYR61 (Figure 5D). We further determined the relationship between CYR61 degradation and its phosphorylation by PLK1. Our results revealed that PLK1-CA caused significant degradation of WT CYR61, whereas it had minimal impact on CYR61 mutants with S167A, S188A, or S167A/S188A (double-mutant, DM) (Figure 5E). In contrast, PLK1-KD did not induce degradation of either WT CYR61 or any of the aforementioned mutants (Figure 5E). These findings were consistently supported by measuring the half-life of WT CYR61, which was considerably shorter compared to that of the three mutants (Figure 5F, G).

Since the degradation of CYR61 by PLK1 predominantly occurs during mitotic exit (Figure 4F, G), we asked if PLK1-mediated phosphorylation is indispensable for CYR61 turnover at this stage. To address this, we ectopically transfected HeLa cells with either WT CYR61 or DM CYR61 and employed nocodazole treatment to synchronize them at prometaphase. Subsequently, we released the cells in CHX-containing medium and measured levels of exogenous CYR61 protein at specific time points. Strikingly, the expression of WT CYR61 significantly decreased at 2 hours post-release, indicating rapid degradation. In contrast, the level of DM CYR61 remained relatively stable, with only slight degradation observed at up to 2.5 hours post-release (Figure 5H, I). Overall, these findings establish that PLK1 directly phosphorylates both the S167 and S188 sites of CYR61, thereby influencing the degradation of CYR61 during mitosis.

### CYR61 is ubiquitinated and degraded in an FBW7-dependent manner

**Figure 6:**
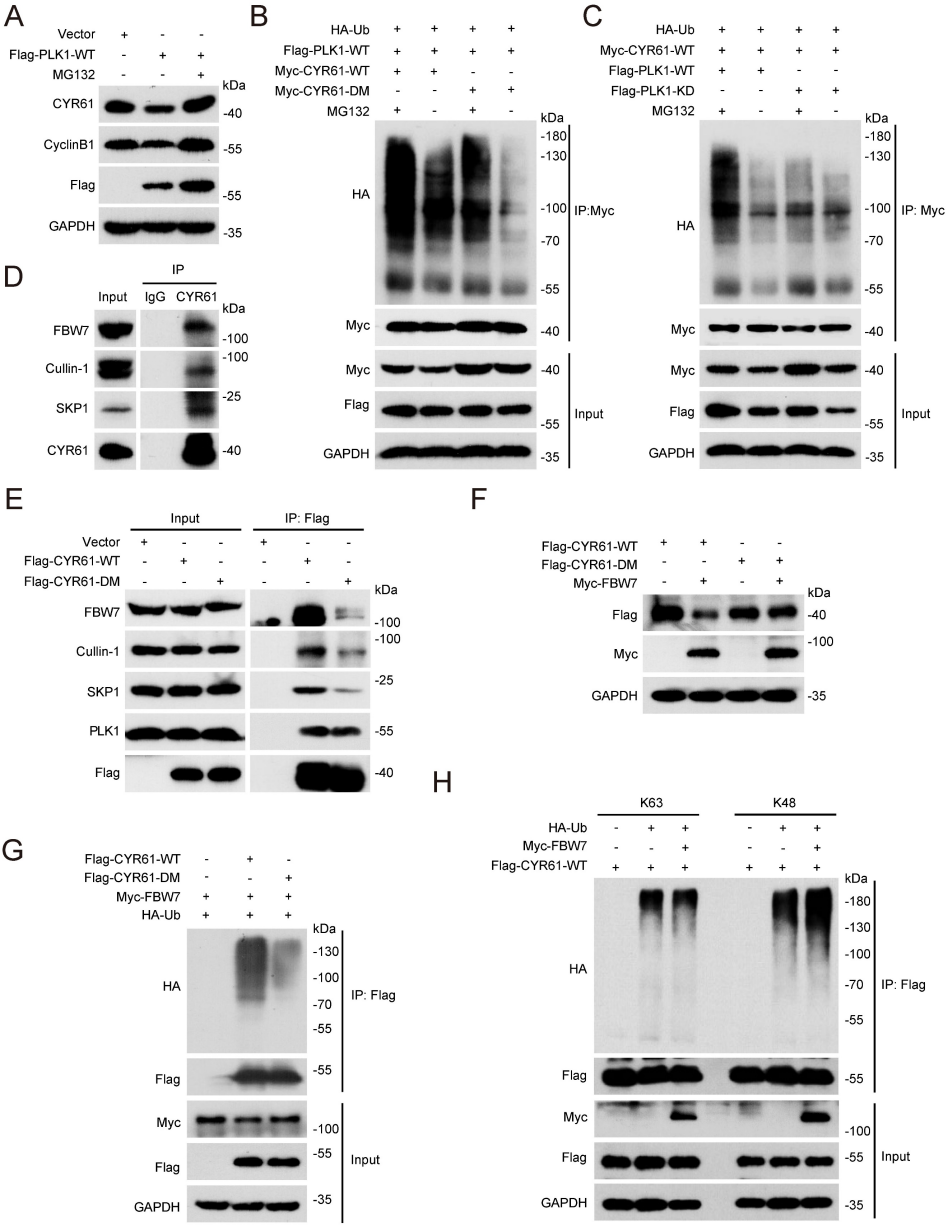
FBW7 facilitates the ubiquitination and subsequent degradation of phosphorylated CYR61. **(A)** HeLa cells transfected with indicated plasmids were treated in the presence or absence of MG132 (10 μM). Subsequently, cells were lysed with RIPA buffer and analyzed by western blotting using indicated antibodies. Biological triplicates were performed and the representative results are presented. **(B and C)** HeLa cells transfected with indicated plasmids were treated in the presence or absence of MG132 (10 μM) and subjected to immunoprecipitation with anti-Myc beads. Subsequently, cell lysates were analyzed by western blotting using indicated antibodies. Biological triplicates were performed and the representative results are presented. **(D)** Cell lysates from HeLa cells were immunoprecipitated with either anti-CYR61 antibody or IgG and were subsequently immunoblotted using indicated antibodies. Biological triplicates were performed and the representative results are presented. **(E)** HeLa cells transfected with indicated plasmids were immunoprecipitated with anti-Flag beads. Subsequently, cell lysates were analyzed by western blotting using indicated antibodies. Biological triplicates were performed and the representative results are presented. **(F)** HeLa cells transfected with indicated plasmids were lysed with RIPA buffer and analyzed by western blotting using indicated antibodies. Biological triplicates were performed and the representative results are presented. **(G and H)** Cell lysates from HeLa cells transfected with indicated plasmids were immunoprecipitated with anti-Flag beads. Subsequently, cell lysates were analyzed by western blotting using indicated antibodies. Biological triplicates were performed and the representative results are presented.

Given that the ubiquitin/proteasome pathway is the fastest known mechanism for irreversibly degrading proteins 42, 43, we investigated whether PLK1 could regulate CYR61 through affecting its ubiquitination. Firstly, we demonstrated that the proteasome inhibitor MG132 effectively reversed the reduction of CYR61 induced by PLK1 (Figure 6A). Moreover, in the presence of ectopically expressed PLK1 and MG132, WT CYR61 was more heavily ubiquitinated than the CYR61-DM mutant (Figure 6B). Additionally, WT PLK1, but not its KD form, significantly elevated the poly-ubiquitination level of CYR61 (Figure 6C). These observations provide compelling evidence that the phosphorylation of CYR61 at both S167 and S188 sites, along with the kinase activity of PLK1, collectively modulate the poly-ubiquitylation of CYR61 and subsequently elicit its degradation by the proteasome.

Next, we wondered which E3 ubiquitin ligase complex is responsible for targeting CYR61. This inquiry was reminiscent of our prior MS findings, which also suggested a potential interaction between CYR61 and CUL1, a core component of the SCF E3 ligase (Figure 1B). Indeed, co-IP assays unveiled distinct interactions among CYR61, CUL1, SKP1, and FBW7 in the immunoprecipitated complex (Figure 6D). Considering that F-box proteins of the SCF complex often recognize substrates when they are properly modified, most cases entailing phosphorylation of the degron motif within the specific substrate 22, we proceeded with a more in-depth investigation into the regulatory relationship between CYR61 and FBW7.

Firstly, we proved that the knockdown of FBW7 had a notable impact on elevating the protein levels of CYR61 across multiple cancer cell lines, including HeLa, H1299, and MDA-MB-231 cells (Figure S4A). Secondly, in the presence of CHX, the half-life of endogenous CYR61 significantly extended in FBW7-depleted cells (Figure S4B, C). Additionally, when compared to the CYR61-DM mutant, the WT CYR61 exhibited a more pronounced binding affinity with each main component of the SCF-FBW7 complex (Figure 6E). Moreover, the expression level of WT CYR61 was noticeably more reduced in the presence of FBW7 compared to that of the DM CYR61 (Figure 6F), which was in parallel with the observation that WT CYR61 underwent more profound ubiquitination by FBW7 (Figure 6G). Altogether, these findings confirm the necessity of phosphorylation at sites S167 and S188 of CYR61 for FBW7 recognition, which subsequently leads to CYR61 proteolysis via the proteasome pathway.

Finally, we determined the type of ubiquitination linkage that FBW7 employs on CYR61. Typically, the K48-linked ubiquitination is associated with proteasome-mediated protein degradation, whereas the K63-linked ubiquitination regulates protein functionality 44. As expected, the level of K48-linked ubiquitination of CYR61 significantly increased in HeLa cells where FBW7 was ectopically overexpressed. In contrast, the K63-linked ubiquitination of CYR61 remained nearly unchanged (Figure 6H). These findings provide further confirmation that CYR61 undergoes K48-linked ubiquitination in a manner contingent upon FBW7.

## Discussion

The cell cycle is a series of processes that orchestrates cell division, leading to the production of two new daughter cells 16. Sustained cell proliferation represents a key hallmark of cancer, which often arises due to deficiencies or malfunctions in the cell cycle regulation system 45. Among different phases of the cell cycle, mitosis stands out for its rigorous control mechanisms, which relies on a network of proteins to ensure the accurate distribution and preservation of genetic material 46-49. An essential player in this process is PLK1, which phosphorylates specific target proteins and governs multiple mitotic activities 50. For instance, PLK1 facilitates mitotic entry by inducing the phosphorylation of phosphatase CDC25C 51. Additionally, PLK1 phosphorylates KNL-1 and Mps1 to regulate the SAC 39. Notably, the expression of PLK1 fluctuates throughout the cell cycle, with low levels during the G1/S transition, a gradual increase during the S phase, and reaching its maximum in the G2/M phase 50.

**Figure 7:**
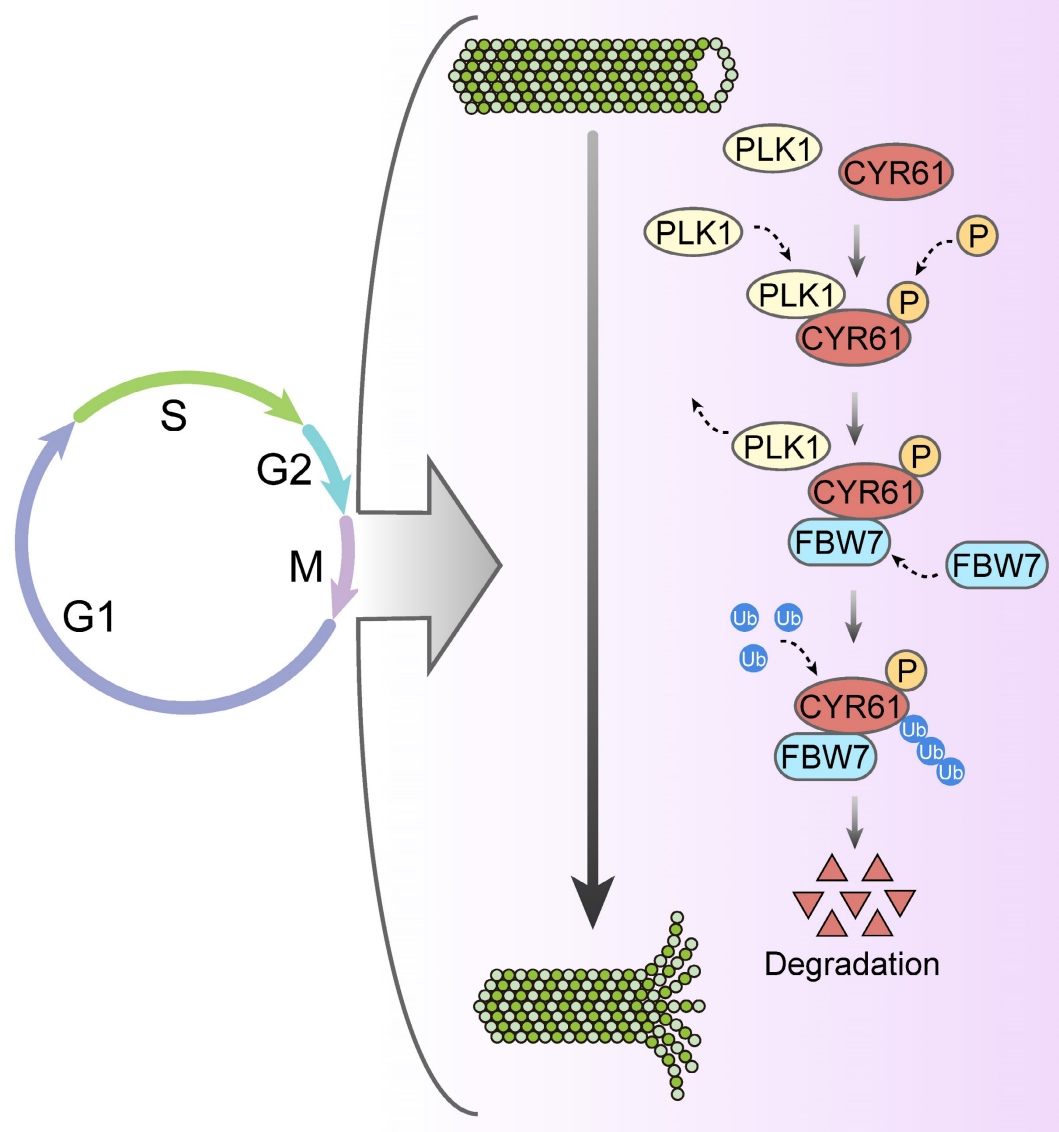
Proposed model for the intracellular role and post-translational regulation of CYR61 during mitosis.

Likewise, our study has uncovered a significant abundance of CYR61 during the mitotic phase (Figure 3A). Intriguingly, our MS results suggested that CYR61 and PLK1 form protein complexes (Figure 1A, B). This discovery prompted us to investigate the collaboration between PLK1 and CYR61 during mitosis. As anticipated, our findings have confirmed that PLK1 indeed interacts with CYR61 and exerts regulatory control over it through phosphorylating sites S167 and S188 (Figures 3-5 and Figure S3). Specifically, the phosphorylation of CYR61 facilitates its recognition by the SCF-FBW7 complex, leading to its precise degradation via the proteasome pathway (Figure 6 and Figure S4). Furthermore, when the normal expression of CYR61 is disrupted, it results in misoriented cell division and mitotic arrest, as depicted in Figure 2 and Figure S2. Thereby, our findings highlight a novel intracellular role for CYR61, which holds significant implications for both cell cycle progression and cellular development (Figure 7).

In this study, we also noted an interaction between CYR61 and tubulin during the process of mitosis (Figure 1D-F), indicating that CYR61 might also serve as an intracellular microtubule-associated protein (MAP). Being integral components of the cytoskeleton, microtubules are built from tubulin heterodimers and participate in various cellular functions, including mitosis and cell division 52. Biochemical studies have shown that MAPs strictly regulate the structure, dynamic behavior, and spatial organization of microtubules. One of the most known MAPs is Tau, which binds to microtubules through evolutionarily conserved residues and stabilizes microtubules by attaching to a hydrophobic pocket situated between tubulin heterodimers 53. Additionally, the phosphorylation of Thr231 and Ser235 on Tau by kinases such as CDK2 has been proved to be crucial for the Tau-mediated microtubule assembly 54. Conversely, impaired interaction between Tau and microtubules plays an important role in the pathology of various neurodegenerative diseases, including Alzheimer's disease 55, 56. MAP9 (microtubule-associated protein 9) is another well-known MAP that is essential for the mitotic apparatus. Studies have revealed that MAP9 is the substrate of kinases such as Aurora A and PLK1 and is recruited to spindle poles through the NEDD1-γ-tubulin pathway 57, 58. Notably, the PLK1-mediated phosphorylation of MAP9 at Ser289 occurs at centrosomes and contributes to the spindle pole stability in a microtubule-dependent manner, underscoring MAP9's role in ensuring faithful chromosome segregation during mitosis.

In our study, we also observed that CYR61 promotes the stability and assembly of microtubules (Figure 1G-I). Moreover, depletion of CYR61 leads to the prolonged duration of each stage of mitosis and results in defective orientation and irregular positioning of the mitotic spindle (Figure 2E-G). We also identified CYR61 as a novel substrate of PLK1 during mitosis, with S167 and S188 being the major phosphorylation sites (Figure 5). However, more intricate mechanisms through which CYR61 interacts with microtubules and promotes microtubule assembly or spindle formation remain poorly elucidated. Does CYR61 impact the stability properties of microtubules in a manner akin to Tau, possibly by affecting microtubule labile domains 59? Could the PLK1-induced phosphorylation of CYR61 at S167 and S188 sites influence CYR61's subcellular localization or its interaction with tubulins, thereby affecting spindle pole organization and integrity similarly to MAP9^57^? Addressing these questions will provide insights into how CYR61 modulates microtubule dynamics and may establish CYR61 as a promising and novel therapeutic target.

In addition, through IP assays and MS analysis, we discovered interactions between CYR61 and other tubulin-associated proteins, such as MAP4, MAP6, and DVL-1, which either promote microtubule assembly or disassembly (Figure S1 and Supplementary Table 1). For instance, prior research has demonstrated that MAP4 enhances microtubule stability and facilitates microtubule polymerization by modulating tubulin partitioning between its monomeric and polymeric form, as well as by influencing tubulin synthesis 60. Given that CYR61 has been shown to stimulate microtubule polymerization *in vitro* (Figure 1I), it is conceivable that CYR61 could impact the stability and dynamics of microtubules via cooperation with MAP4 or other MAPs. Further investigation into the regulatory signaling pathways linking CYR61 and other MAPs will enhance our understanding of CYR61's roles during mitosis.

Our study also has several other limitations that should be acknowledged. Firstly, we did not investigate the role of CYR61 in cell cycle phases beyond mitosis. Given that CDKs are essential for driving each cell cycle phase 61, future research is required to delve into the interplay between CYR61 and CDKs to obtain a more comprehensive understanding of CYR61's function throughout the cell cycle process. Secondly, despite HeLa cells are widely recognized and extensively used as a model in biological research, it is imperative to validate the findings regarding CYR61's functions in this study using additional models, including immortalized normal cell lines. Lastly, it's worth nothing that previous reports have suggested that CYR61 is involved in signaling pathways mediating paclitaxel resistance in breast cancer cells 25. Since CYR61 functions as a mitotic-regulating MAP, it is plausible that CYR61 may also be associated with microtubule-targeting agent like paclitaxel. Therefore, we hypothesize that the dynamic expression of CYR61 in different cell cycle phases could also potentially contribute to paclitaxel resistance. Further studies are needed to explore the roles of CYR61 in mediating paclitaxel resistance thoroughly.

## Materials and methods

### Cell culture

HeLa, MCF-7, HEK293T, H1299 and MDA-MB-231 cells were obtained from the ATCC (Manassas, VA, USA) and cultured in Dulbecco's modified Eagle's medium (DMEM; Life Technologies, Grand Island, NY, USA) supplemented with 1% penicillin-streptomycin and 10% fetal bovine serum (FBS, Life Technologies) with 5% CO_2_ at 37 °C.

### Bacteria strains and growth conditions

All *E. coli* strains used in this study were cultured in liquid LuriaBertani (LB) broth at 37°C with shaking at 200 rpm.

### Plasmid construction and transfection

Full-length cDNAs encoding *CYR61* and *PLK1* were derived through PCR amplification from human HEK293 cells and were subsequently cloned into the pcDNA3.1 vector with indicated tag sequences. Mutations were introduced using the Takara MutanBEST Kit (Takara, Shiga, Japan; D40) using the following primers:

CYR61-S167A-Forward: 5'-GCTATCAAGGACCCCATGGAGG-3'; CYR61-S167A-Reverse: 5'-ATCCTCGTCACAGACCCACTCC-3'; CYR61-S188A-Forward: 5' -GCCGAGGTGGAGTTGACGAGAA-3'; CYR61-S188A-Reverse: 5'-GGCATCGAATCCCAGCTCCTT-3'.

For CRISPR‒Cas9 knockout of human *CYR61* in HeLa cells, the following small guide RNA (sgRNA) targeting sequences were used:

sg*CYR61*#1: CGGGCTGGGGCGGTACGCGC;

sg*CYR61*#2: ACGTAAAGAAGAATTATCCG.

HeLa cells were infected with the lentivirus supernatant, followed by selection with media containing puromycin (2 µg/mL) for 72 h. Plasmid transfections were performed using Lipofectamine 3000 (Life Technologies). siRNA transfections were performed using Lipofectamine RNAiMAX reagent (Life Technologies) according to its protocol. The sequences of the indicated siRNAs are as follows:

si-*CYR61*-1: AATGAATTGATTGCAGTTGGA;

si-*CYR61*-2: GGTGGAGTTGACGAGAAAC;

si-*PLK1*-1: AGAUCACUCUCCUCAACUAUU;

si-*PLK1*-2: GGACATGGCTGTGAATCAG;

si-*PLK1*-3: CGGCCTCATGCGCACATCC;

si-*PLK1*-4: CTGGTAGTACTAGTTCACCTA;

si-*FBW7*: GCATATGATTTTATGGTAA;

### Immunoprecipitation and western blotting

Immunoprecipitation and western blotting were performed as described. Briefly, cells were lysed in RIPA buffer containing protease and phosphatase inhibitors (Bimake, Shanghai, China). Clarified lysates were then incubated with either indicated antibodies or beads overnight at 4 °C, followed by washing three times with NETN buffer (nuclear and cytoplasmic extraction buffer: 20 × 10^-3^ M Tris-HCl, 100 × 10^-3^ M NaCl, 1 × 10^-3^ M EDTA, 0.5% Nonidet P-40, and protease and phosphatase inhibitors). Finally, samples were boiled in 2 × SDS loading buffer, resolved using SDS-PAGE, and transferred to polyvinylidene fluoride (PVDF) membranes followed by blocking with 5% skim milk in phosphate-buffered saline-Tween 20 (PBST) and incubation with indicated antibodies.

### Antibodies and reagents

Human anti-CYR61 (26689-1-AP), anti-α-Tubulin (11224-1-AP), anti-DVL-1 (27384-1-AP), anti-DVL-2 (67105-1-AP), anti-DVL-3 (13444-1-AP), anti-Cyclin D1 (60186-1-Ig), anti-PLK1 (10305-1-AP), anti-Cyclin B1 (28603-1-AP), and anti-acethyl-tubulin (66200-1-Ig) antibodies were purchased from Proteintech. Human anti-Myc (#2276), anti-Phospho-Histone H3 (Ser10) (#53348), anti-Cullin-1 (#4995), anti-SKP1 (#12248), anti-His (#9991), anti-GST (#2622), anti-Phospho-PLK1 (Thr210) (#5472), and anti-rabbit IgG (#7074) antibodies were purchased from Cell Signaling Technology. Human anti-Aurora B (611082), anti-β-Tubulin (556321), anti-P150 (610473), and anti-NUMA (610561) antibodies were purchased from BD Bioscience. Human anti-DIC1/2 (sc-66866) antibody was purchased from Santa Cruz Biotechnology. Human anti-FBW7 (ab81256) antibody was purchased from Abcam. Human anti-GAPDH (bs-2188R) antibody was purchased from Bioss. Human anti-Flag (#F1804) antibody was purchased from Sigma-Aldrich. The proteasome inhibitor MG132 was purchased from Selleck Chemicals. Cycloheximide (CHX), nocodazole, and paclitaxel were purchased from Sigma Chemical. BI2536 and AZD1152 were purchased from Selleck Chemicals. λ-PPase was purchased from New England BioLabs.

### Immunofluorescence staining

Immunofluorescence was carried out as described. Briefly, cells grown on glass coverslips were treated with indicated drugs and then washed once with PBS, followed by pre-extraction with buffer containing 0.5% Triton X-100 for 3 min and fixation with 3% paraformaldehyde for 10 min at room temperature. Cells were then incubated with indicated antibodies at 4 °C overnight, washed with PBS three times, and stained with secondary antibodies at room temperature for 1 h. Finally, cells were stained with 4′,6-diamidino-2-phenylindole (DAPI) to visualize nuclear DNA and were then subjected to fluorescence microscopy analysis.

### Tubulin polymerization assay

Tubulin polymerization assay was measured by the Tubulin Polymerization Assay Kit (cytoskeleton cat. #BK006P) following the manufacturer's instructions.

### Mass spectrometry analysis

CYR61 Co-IP samples were digested in 50 mM ammonium bicarbonate buffer overnight at 37℃ with trypsin (Promega, Madison, WI). Peptides were analyzed by Thermo Scientific Orbitrap Fusion mass spectrometer. The Mass Spectrometry analysis results are provided in Supplementary Table 1.

### GST-PLK1 pulldown assay

The His-CYR61-WT and GST-PLK1-WT were both purified from *E. coli*. Then, the fusion protein His-CYR61-WT was incubated with either GST or GST-PLK1-WT at 4℃ overnight. Subsequently, anti-GST beads were added into the system, followed by separation and western blotting analysis.

### Cell synchronization

HeLa cells were synchronized at the G1/S boundary by performing a double thymidine block-and-release treatment. Cells were released into normal media at the following time points: 3 h (S-phase), 6 h (G2-phase), 12 h with incubation of 100 ng /ml nocodazole (M-phase) and 11 h (G1-phase). Cells were harvested and subjected to indicated experiments.

### Statistical analysis

GraphPad Prism version 9 was used to analyze the data. All experiments subjected to statistical analysis were repeated at least two times. Results were presented as mean ± standard error of the mean (SEM) and statistical analyses were made using the Student's t-test.

## Supplementary Material

Supplementary tables.
